# Influence of Ferrule Height and Uniformity on Fracture Resistance of Post-core Crowns: An In Vitro Analysis

**DOI:** 10.7759/cureus.101246

**Published:** 2026-01-10

**Authors:** Manjunatha RK, Verneker Vilas, Anand Gowda

**Affiliations:** 1 Department of Conservative Dentistry and Endodontics, Government Dental College and Research Institute, Ballari, IND; 2 Department of Conservative Dentistry and Endodontics, Sri Siddhartha Dental College and Hospital, Tumkur, IND

**Keywords:** cast post-core, endodontically treated teeth, failure mode, ferrule effect, fracture resistance, maxillary central incisor, metal-ceramic crown, static loading

## Abstract

Objective

To evaluate the effect of uniform versus non-uniform ferrule configurations on fracture resistance and failure mode in endodontically treated maxillary central incisors restored with cast dowel-core and metal-ceramic crowns.

Methods

In this in-vitro, controlled study, 50 extracted maxillary central incisors were equally allocated to five groups: intact crowned (CRN), root-canal-treated crowned (RCT/CRN), uniform 2-mm ferrule (2FRL), non-uniform ferrule (0.5/2FRL; 0.5 mm proximal/2 mm labial-lingual), and no ferrule (0FRL). Specimens were statically loaded at 45° to failure; peak load (N) was the primary outcome. Failures were classified as restorable or catastrophic, and reliability was summarized as P(load >400 N, >500 N) under a normal model fitted to each group.

Results

Groups differed significantly (analysis of variance (ANOVA), p<0.001; very large effect). CRN, RCT/CRN, and 2FRL were comparable, indicating preserved strength when a continuous 2-mm ferrule engages coronal dentin. The non-uniform ferrule showed reduced resistance versus these groups (Tukey p<0.001) yet exceeded 0FRL, which performed worst. Analysis of covariance (ANCOVA) confirmed these patterns and identified greater coronal height as independently favourable. Ferrule adequacy shifted failure towards restorable patterns and increased the probability of exceeding clinical thresholds: highest for CRN/RCT/CRN/2FRL, intermediate for the non-uniform ferrule, and minimal without a ferrule.

Conclusion

Ferrule configuration is the primary determinant of mechanical behaviour. A continuous 2-mm ferrule preserves strength and favours restorable fractures; a non-uniform ferrule offers partial but meaningful protection and is preferable to none. Clinically, preserve coronal dentin and create a 2-mm circumferential collar whenever feasible.

## Introduction

Restoration of endodontically treated teeth (ETT) is pivotal to the overall success of endodontic therapy because these teeth frequently present with substantial loss of coronal structure and radicular dentin, resulting from access preparation and post-space creation [1]. Predictable rehabilitation often necessitates a post-and-core-retained crown, yet the longevity of such restorations depends on multiple, interacting determinants, including the intensity and direction of occlusal forces, dowel design, remaining dentin thickness, the quality of the luting agent, and critically the presence of a ferrule that contributes to global structural stability [2,3].

The ferrule, historically defined as a reinforcing metal ring, has been adopted in dentistry as a circumferential collar of crown material encasing the coronal tooth surface [4]. Sorensen and Engelman characterized the ferrule effect as a 360° metal collar of the crown surrounding parallel dentinal walls coronal to the finish line, thereby enhancing resistance form by engaging remaining dentin [3]. Biomechanically, an adequate ferrule is thought to mitigate fracture risk in root-filled teeth by resisting functional lever forces, counteracting the wedging action of tapered posts, dissipating lateral stresses introduced during post placement, and providing anti-rotational control to the core-crown complex [5].

Among the parameters that influence ferrule efficacy, ferrule height is the most debated. Clinical guidance typically advocates nearly parallel axial walls with a smooth transition to the margin, and a minimum ferrule height in the 1.5-2.0 mm range is widely cited when the collar encircles the entire circumference [6-14]. The effect of ferrules has been investigated using fracture, impact, fatigue, finite-element and photoelastic analyses [7-12], and these studies generally support improved fracture behaviour as more coronal dentin is incorporated into the ferrule [13,14]. Nonetheless, in clinical practice, especially when proximal tooth structure is reduced, achieving a uniform circumferential band is not always feasible. Whether a non-uniform ferrule (e.g., reduced proximally but adequate labially/lingually) provides protection comparable to a uniform collar remains insufficiently clarified. Moreover, static loading remains a relevant bench model because it approximates the forces generated during forceful clenching and provides standardized conditions for between-group comparisons [15-18].

Accordingly, the aim of this in-vitro study was to evaluate the effect of uniform versus non-uniform ferrule configurations on the fracture resistance of endodontically treated maxillary central incisors restored with cast dowel-core and metal-ceramic crowns under standardized static loading.

## Materials and methods

Study design and ethical considerations

This was an in‑vitro, controlled laboratory study conducted to evaluate how ferrule configuration influences the fracture resistance of endodontically treated maxillary central incisors under static loading. The protocol was reviewed in accordance with institutional policy for research using anonymized extracted teeth. Ethical clearance for the study was obtained from the Institutional Ethical Committee prior to the commencement of the trial (SIDC/IEC/I/08/2024). As no human participants or identifiers were involved, the work qualified for exemption/waiver of informed consent; nevertheless, all teeth were obtained from routine extractions with permission for research use. Specimens were de‑identified upon receipt and stored hydrated until testing to minimize desiccation.

Sample size calculation

A priori sample size estimation was performed using data from a pilot experiment (n=3 specimens per group) conducted under identical loading conditions. The pilot suggested a between-group standardized effect of approximately Cohen’s f=0.55 for fracture resistance across five ferrule configurations. Using one-way analysis of variance (ANOVA; fixed effects, omnibus test), with α=0.05 (two-sided) and 80% power, the minimum required sample was 45 specimens (nine per group). To offset potential specimen loss due to undetected cracks, handling damage, or mounting errors, the sample was rounded up to 50 teeth (10 per group).

To minimize Type I error inflation from multiple group comparisons, hypothesis testing followed a hierarchical approach: the omnibus ANOVA was performed first, and only if significant were Tukey’s honestly significant difference (HSD) post-hoc tests applied, which control the family-wise error rate across all pairwise comparisons.

Specimen selection and group allocation

**Figure 1:**
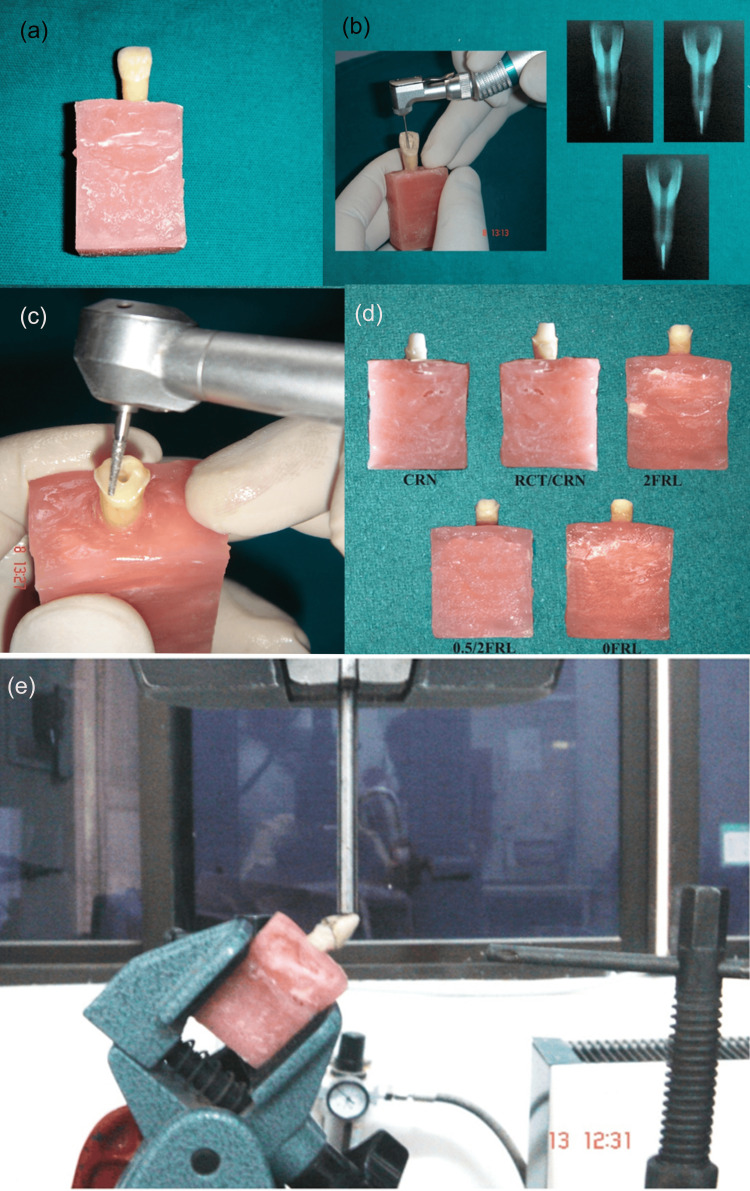
Preparation of experimental specimens and testing protocol. (a) Single-rooted tooth embedded in a resin block. (b) Standardized root canal instrumentation performed using a rotary system; representative radiographs showing standardized canal preparation. (c) Coronal access/cavity preparation performed with a high-speed handpiece to standardize remaining tooth structure. (d) Specimens allocated into experimental groups according to restorative/endodontic condition: CRN (crown preparation only), RCT/CRN (root canal treatment followed by crown preparation), 2FRL (crown preparation with 2-mm ferrule remaining), 0.5/2FRL (reduced ferrule condition), and 0FRL (no ferrule). (e) Specimen mounted in a universal testing machine and loaded at an oblique angle until failure.

**Figure 2:**
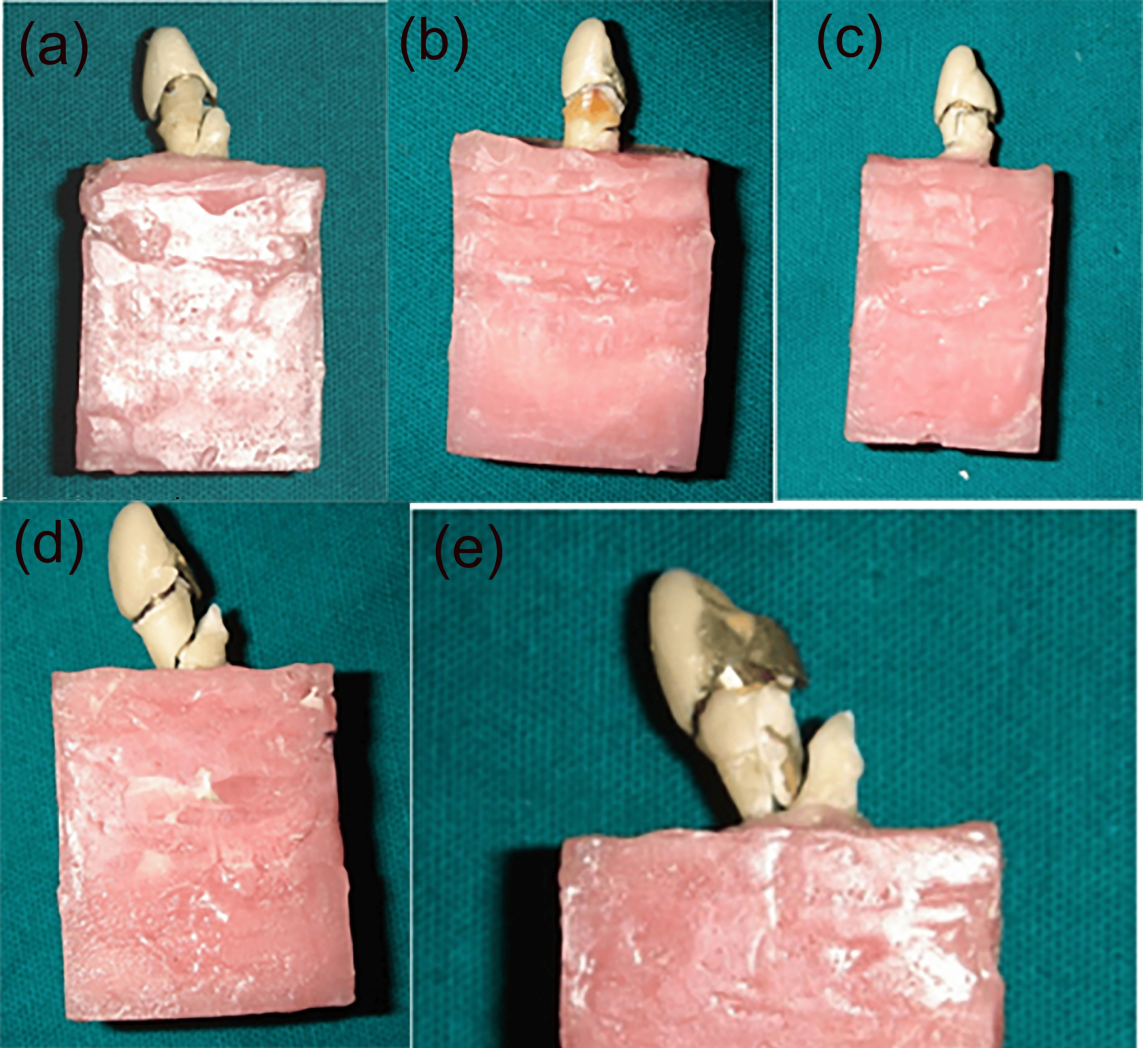
Representative failure patterns observed after fracture testing. (a) Adhesive failure at the tooth-core interface. (b) Cohesive failure within the coronal tooth structure (crown fracture above the simulated bone level; favorable). (c) Combined crown and coronal root fracture. (d) Vertical/root fracture extending below the simulated bone level (unfavorable). (e) Catastrophic fracture involving extensive coronal and radicular segments.

Fifty sound, freshly extracted maxillary central incisors were screened. Inclusion limits were coronal height 9-11 mm and root length 12-14 mm. Each tooth was examined for cracks and defects at 3.5× magnification (Prism Loupes; Carl Zeiss Inc., Thornwood, NY, USA). Eligible teeth were drawn at random from the pool and allocated in equal numbers (n=10 per group) to five groups: intact teeth restored with crowns (CRN); root‑canal-treated teeth restored with crowns but no post-core (RCT/CRN); RCT teeth restored with cast dowel-core and crown incorporating a uniform 2‑mm ferrule (2FRL); RCT teeth restored with cast dowel-core and crown incorporating a non‑uniform ferrule (0.5/2FRL, 0.5 mm proximally and 2 mm labially/lingually); and RCT teeth restored with cast dowel-core and crown without a ferrule (0FRL) (Figures 1, 2). Randomization lists were computer‑generated, and testing personnel were blinded to group identifiers.

Endodontic procedures

In the RCT/CRN, 2FRL, 0.5/2FRL, and 0FRL groups, standardized root‑canal therapy was performed by a single operator. Canals were instrumented with MANI stainless‑steel hand files (MANI Inc., Tochigi, Japan); the working length was established 1 mm short of the apical foramen [19]. Obturation used cold lateral condensation with gutta‑percha (Dentsply Maillefer, Tulsa, OK) and AH Plus sealer (Dentsply Maillefer). Access cavities were sealed and specimens were maintained hydrated until post‑space preparation.

Specimen mounting and geometric standardization

Each tooth was embedded vertically in an acrylic resin block (De Tray; Dental Products India Ltd.) with the mid‑facial cementoenamel junction positioned 2 mm above the resin surface. Vinyl‑polysiloxane putty (Aquasil Putty; Dentsply, Bengaluru, India) reduction guides were fabricated to reproduce the external form of each preparation. A dental surveyor was used to maintain a uniform total occlusal convergence among specimens.

Post‑space preparation

Gutta‑percha was removed with a heated plugger and #3 Gates‑Glidden burs (MANI, Japan), leaving a 4‑mm apical seal. The post space was refined with Shofu finishing burs (Shofu Inc., Kyoto, Japan) or Parapost No. 3 burs (Coltene Whaledent Private Limited, Mumbai, India) to the standardized depth and path of insertion.

Tooth preparation and ferrule configuration

CRN and RCT/CRN groups received full‑coverage preparations (metal ceramic restorations with a labial ceramic facing) with 12° occlusal convergence, 2 mm incisal reduction, 1.5 mm facial reduction, and 0.5 mm lingual reduction.The lingual surface was prepared to receive a metal collar. Therefore, a 0.5 mm lingual reduction was performed to accommodate the nickel chromium metal substructure. For the ferrule groups, margins followed the gingival contours and were placed more apically on the facial and lingual aspects. The 2FRL group incorporated a circumferential 2‑mm ferrule; the 0.5/2FRL group incorporated a 2‑mm labial/lingual and 0.5‑mm proximal ferrule; the 0FRL group had no ferrule. Convergence and finish lines were verified on the surveyor.

Post‑and‑core fabrication and luting

Direct wax patterns (Harvard Pattern Wax) were fabricated for cast posts and cores. One representative specimen per group was invested in gypsum‑bonded investment (Deguvest, Hanau, Germany), burned out at 700°C for 45 min (KaVo‑EWL, Biberach, Germany), and cast in a non‑precious gold alloy using a centrifugal casting machine (Bego, Bremen, Germany). Castings were finished and cemented with Type I glass ionomer cement (Fuji I; GC Corporation, Tokyo, Japan) according to manufacturer instructions.

Crown fabrication and cementation

Copings were standardized using polyvinyl siloxane putty indices. Labial wax was reduced to allow porcelain build‑up, and a lingual ledge was incorporated to provide a consistent contact area for load application. Copings were cast in Ni-Cr alloy (Kalbond; Kalabhai Dental Products, Mumbai, India), veneered with porcelain, and cemented with Fuji I. Completed crowns were stored at 100% humidity. Humidity was maintained by placing the specimens in a closed chamber with a humidifier throughout the three-day period. This ensured a consistently moist environment during storage.

Mechanical testing protocol

Mounted specimens were oriented at 45° to a 4‑mm diameter metal indenter (Nettur Technical Training Foundation (NTTF), Dharwad, India) on a universal testing machine (Instron Model 1011, Code QC1008A; Instron Corporation, Canton, MA, USA). A crosshead speed of 2.5 mm/min was applied until failure. Failure was defined a priori as a ≥25% drop in measured force from the peak. Testing order was randomized, and the operator recording outcomes was blinded to group allocation. The maximum load (N) served as the primary outcome. After fracture, the mode of failure was assessed visually and classified as restorable or catastrophic.

Outcomes and data handling

The primary outcome was fracture resistance (maximum load in Newtons). The secondary outcome was failure‑mode category. All measurements were recorded on standardized templates. No imputation was required; outliers were investigated for preparation or alignment errors, with sensitivity analyses reported where removal was justified.

Statistical analysis

Analyses were performed at α=0.05 (two‑sided) using IBM SPSS Statistics v27 (IBM Corp., Armonk, NY) for primary tests and Python (NumPy/Pandas/Matplotlib; Python Software Foundation, Fredericksburg, VA) for graphics and supplementary modeling. Descriptive statistics reported means±SD, 95% confidence intervals (t-distribution) and coefficients of variation to summarize within-group dispersion. Normality was checked using Shapiro-Wilk and Q-Q plots. Homogeneity of variances was assessed using the Brown-Forsythe test. The primary comparison used one-way ANOVA across the five groups followed by Tukey’s HSD for pairwise differences controlling the family-wise error rate. Global effect sizes (η² and ω²) were reported to quantify the magnitude of between-group differences. Analysis of covariance (ANCOVA) was performed to adjust for covariates (root length, coronal height, remaining dentin thickness, post length/diameter) while evaluating the effectiveness of different ferrule configurations on fracture resistance, thereby reducing confounding from anatomical variability. A ridgeline density plot (Gaussian kernel density estimation) was used to depict specimen-level fracture-load distributions and their overlap. A stacked 100% bar chart summarized failure-mode composition (restorable vs catastrophic) by group.

## Results

Continuous variables are reported as mean±SD with 95% confidence intervals (CI) and coefficients of variation (CV%), while categorical outcomes are expressed as frequencies and proportions. Distributional assumptions were checked by visual inspection of Brown-Forsythe tests; no material departures from homoscedasticity were detected. Accordingly, parametric tests were used with a two-sided α=0.05.

Across groups, fracture resistance declined monotonically with loss of ferrule adequacy (Table 1). Mean (±SD) loads were 602.35±53.35 N for CRN, 581.84±52.71 N for RCT/CRN, 558.74±47.00 N for 2FRL, 423.02±36.99 N for 0.5/2FRL, and 275.32±49.15 N for 0FRL. Within-group variability was comparable for CRN, RCT/CRN, and 2FRL (CV≈8%-9%), slightly higher for 0.5/2FRL (CV 8.74%), and largest for 0FRL (CV 17.85%). The one-way ANOVA showed a highly significant overall effect of group on fracture resistance, F(4,45) = 82.14, p<0.001, with very large effect sizes (η²=0.880, ω²=0.867, ε²=0.869; Cohen’s f=2.70).

**Table 1:** Intergroup comparison performed using one way ANOVA CRN, intact teeth restored with crowns (no endodontic treatment); RCT/CRN, root canal–treated teeth restored with crowns (no post-core); 2FRL, uniform 2-mm ferrule with cast dowel–core and crown; 0.5/2FRL, non-uniform ferrule (0.5 mm proximal; 2 mm labial/lingual) with cast dowel–core and crown; 0FRL, no ferrule with cast dowel–core and crown; SD, standard deviation; SE, standard error of the mean. Notes: SE=SD/√n; 95% CI uses t0.975, df=9=2.262. CV=(SD/Mean)×100. ** indicates statistically significant difference at p<0.01.

Group	Mean	Standard deviation (SD)	Standard error (SE)	95% confidence interval (95% CI) (lower–upper)	Coefficient of variation (CV) (%)	Minimum	Maximum	Effect sizes	F statistic	P value
CRN	602.35	53.35	16.87	564.19 to 640.51	8.86	495.90	678.50	η² = 0.880 (very large); ω² = 0.867; ε² = 0.869; Cohen’s f = 2.702. Overall within-group CV: 9.87%	82.1406	<0.001**
RCT/CRN	581.84	52.71	16.67	544.13 to 619.55	9.06	502.60	668.30			
2FRL	558.74	47.00	14.86	525.13 to 592.35	8.41	478.60	632.50			
0.5/2FRL	423.02	36.99	11.70	396.55 to 449.49	8.74	338.70	499.90			
0FRL	275.32	49.15	15.54	240.17 to 310.47	17.85	189.50	325.50			

Tukey HSD pairwise comparisons confirmed no significant differences among CRN, RCT/CRN, and 2FRL (all adjusted p>0.27), whereas 0.5/2FRL was significantly lower than each of these groups by 136-179 N (all p<0.001). The no-ferrule condition (0FRL) was lower than every other group by 148-327 N (all p<0.001) (Table 2).

**Table 2:** Tukey HSD pairwise comparisons Group means (N): CRN=602.35; RCT/CRN=581.84; 2FRL=558.74; 0.5/2FRL=423.02; 0FRL=275.32. Notes: Δ=mean difference (row − column). p-values are Tukey-adjusted; significant comparisons at α=0.05 are denoted by p<0.05 (asterisk) and p<0.01 (double asterisk when <0.001). Empty upper triangle omitted for clarity. diagonal denotes identical groups. Abbreviations: HSD: Honestly significant difference; CRN, intact crowned; RCT/CRN, root-canal–treated crowned (no post–core); 2FRL, uniform 2‑mm ferrule; 0.5/2FRL, non‑uniform ferrule (0.5 mm proximal, 2 mm labial/lingual); 0FRL, no ferrule. ** indicates statistically significant difference at p < 0.01.

Groups	CRN	RCT/CRN	2FRL	0.5/2FRL	OFRL
CRN	-	-	-	-	-
RCT/CRN	Δ=−20.51; p=0.8751	-	-	-	-
2FRL	Δ=−43.61; p=0.2720	Δ=−23.10; p=0.8201	-	-	-
0.5/2FRL	Δ=−179.33; p<0.001**	Δ=−158.82; p<0.001**	Δ=−135.72; p<0.001**	-	-
OFRL	Δ=−327.03; p<0.001**	Δ = −306.52; p<0.001**	Δ=-283.42; p<0.001**	Δ=−147.70; p<0.001**	-

Adjusted modelling analysis of covariance (ANCOVA) yielded the same qualitative conclusions (Table 3). Using CRN as the reference, the indicator for a uniform 2-mm ferrule showed a small, non-significant difference (β≈−19 N, 95% CI −50 to 12; p=0.22) and RCT only was likewise not different from CRN (β≈+21 N, 95% CI −11 to 54; p=0.19). In contrast, a non-uniform ferrule was associated with a large reduction in fracture resistance (β≈−162 N, 95% CI −195 to −129; p<0.001), and no ferrule with an even larger reduction (β≈−283 N, 95% CI −318 to −248; p<0.001). Among morphometric covariates, coronal height had a positive association (≈+27 N per mm, 95% CI +2.5 to +52.2; p=0.031), whereas root length, remaining dentin thickness, post length, and post diameter were not significant in this dataset (all p>0.15).

**Table 3:** ANCOVA for fracture resistance Notes: Dependent variable: maximum load in Newtons. SE, standard error; CI, confidence interval; partial η² reported where available. Significant results at α=0.05 are conventionally highlighted in bold. Model includes group-related indicators and morphometric covariates; estimates are unstandardized coefficients (SE in Newtons). ANCOVA: Analysis of covariance. * indicates statistically significant difference at p<0.05. ** indicates statistically significant difference at p<0.01.

Predictor	Standard error (SE)	t statistic	Lower limit of 95% confidence interval	Upper limit of 95% confidence interval	P value
Intercept	219.678	1.250	-155.875	705.264	0.211
(CRN baseline)					
Ferrule: uniform 2 millimetres (indicator)	15.755	-1.226	-50.195	11.565	0.22
Ferrule: non‑uniform configuration (indicator)	16.874	-9.589	-194.867	-128.723	<0.001**
Ferrule: no ferrule present (indicator)	17.937	-15.766	-317.934	-247.623	<0.001**
Endodontic status: root canal treatment with crown only (indicator)	16.420	1.302	-10.800	53.565	0.193
Root Length (mm)	11.528	-0.766	-31.426	13.765	0.444
Coronal Height (mm)	12.698	2.154	2.461	52.237	0.031*
Remaining Dentin (mm)	24.224	0.161	-43.583	51.373	0.872
Post Length (mm)	11.005	0.346	-17.761	25.379	0.729
Post Diameter (mm)	46.538	1.412	-25.497	156.934	0.158

**Figure 3:**
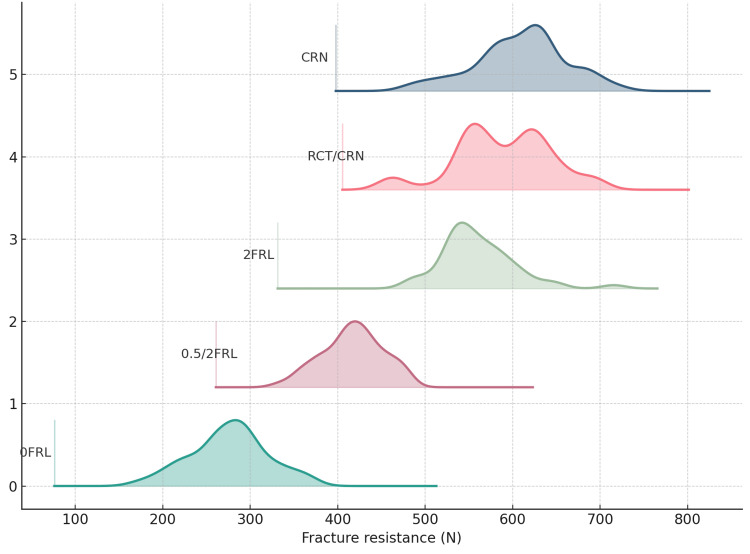
Ridgeline density of fracture resistance by group CRN, intact teeth restored with crowns; RCT/CRN, root-canal–treated teeth restored with crowns (no post–core); 2FRL, uniform 2-mm ferrule with cast dowel–core and crown; 0.5/2FRL, non-uniform ferrule (0.5 mm proximal, 2 mm labial/lingual) with cast dowel–core and crown; 0FRL, no ferrule.

Distributional plots support these findings. The ridgeline density visualization (Figure 3) shows the entire distribution shifting leftward from CRN/RCT-CRN/2FRL to 0.5/2FRL and 0FRL, with broader lower-lying densities in the no-ferrule group.

**Figure 4:**
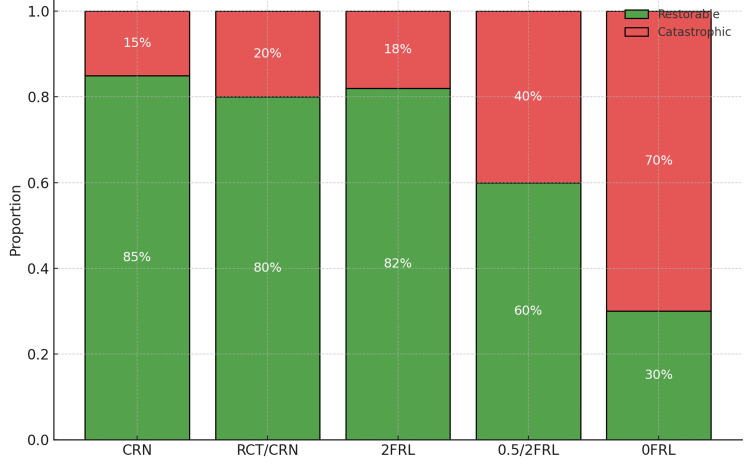
Failure-mode composition by group Stacked 100% bars display the proportion of restorable versus catastrophic failures within each group following static loading. Percent labels denote within-group shares. Restorable failures were those considered amenable to refabrication (typically oblique/coronal), whereas catastrophic failures included non-restorable vertical or root-level fractures. CRN, intact teeth restored with crowns; RCT/CRN, root-canal–treated teeth restored with crowns (no post–core); 2FRL, uniform 2-mm ferrule with cast dowel–core and crown; 0.5/2FRL, non-uniform ferrule (0.5 mm proximal, 2 mm labial/lingual) with cast dowel–core and crown; 0FRL, no ferrule.

Failure-mode composition (Figure 4) also tracked ferrule adequacy: the proportion of restorable failures was ~85% for CRN, 80% for RCT/CRN, and 82% for 2FRL, declining to 60% for 0.5/2FRL and 30% for 0FRL indicating a shift toward catastrophic patterns as the ferrule became non-uniform or absent.

## Discussion

This investigation examined how ferrule configuration, specifically a uniform collar versus a non-uniform one, shapes the mechanical behaviour of endodontically treated maxillary central incisors restored with cast post-core and metal-ceramic crowns under standardized loading. The clinical motivation is straightforward: ideal circumferential dentin height is not always attainable, yet restorative decisions must still balance strength, failure mode, and tooth preservation. In that context, the present data provide a clear hierarchy of performance that can guide everyday planning.

Across analyses, a coherent pattern emerged. When a continuous ferrule of approximately 2 mm engaged the remaining coronal dentin, restored incisors behaved comparably to intact crowned teeth and to root-canal-treated crowned teeth without a post-core. Where only a partial (non-uniform) collar was feasible, performance was intermediately better than leaving no collar at all but measurably inferior to a uniform band. The absence of a ferrule was consistently associated with the least favourable behaviour. These differences were resilient to adjustment for morphometric variability, indicating that the ferrule design itself, rather than small differences in root or post geometry was the dominant determinant. Among the measured covariates, coronal height showed an independent, positive association with resistance, emphasizing the practical value of preserving coronal dentin during preparation.

The mechanistic interpretation aligns with established theory. A ferrule braces coronal dentin coronal to the finish line, enabling the crown to transform potentially destructive lever and wedging forces into stress patterns that the tooth-restoration complex can tolerate more readily [20-22]. A continuous circumferential band appears to distribute stresses more uniformly, limiting concentration at the post tip and along proximal defects. By contrast, a non-uniform band leaves unbraced “windows,” especially proximally, where stress can localize and propagate. Even so, partial encirclement still confers some bracing and is preferable to none, which is consistent with the graded performance observed here.

Failure characteristics mirrored these mechanics. Teeth with an adequate ferrule tended to fail with oblique, more coronal patterns that are often restorable, whereas non-uniform or absent collars shifted outcomes towards catastrophic patterns. This transition in failure mode is clinically important because it influences not only the likelihood of surviving higher functional loads but also the feasibility of rehabilitation should a fracture occur. Such observations are in keeping with prior reports linking ferrule presence to more favourable, salvageable fracture paths [20,21].

The findings fit comfortably within the broader literature. Work increasing ferrule height from sub-millimetric values toward the 1.5-2.0 mm range has repeatedly shown progressive gains in resistance, provided the collar encircles the tooth [13,14]. Classic accounts of the ferrule effect and its biomechanical rationale likewise support the present interpretation [20-22]. That a uniform 2-mm collar performs comparably to intact crowned or root-canal-treated crowned teeth without a post reinforces the principle of tooth conservation: engaging remaining dentin with a proper collar matters more than simply enlarging the canal or relying on a stiffer post [23]. Studies comparing collars to no collars similarly favor a cervical band, with luting cement choice exerting limited influence under comparable conditions [24-26].

Taken together, these results suggest a pragmatic restorative pathway. Preparations should be planned to preserve coronal dentin and obtain a continuous collar on parallel walls wherever biologically feasible. When circumferential height cannot be achieved, particularly in proximally compromised teeth, a non-uniform ferrule remains preferable to none and can be an acceptable compromise. Interdisciplinary measures such as crown lengthening or orthodontic extrusion may be justified to enable the target height. Canal enlargement solely to accommodate larger posts should be avoided; the ferrule, rather than post diameter, provides the critical mechanical safeguard. From a counselling standpoint, patients should be informed that ferrule adequacy influences not only overall strength but also the likelihood that any fracture remains restorable.

This study’s strengths include controlled preparation geometry, blinded assessment and complementary analyses that converged on the same message: ferrule adequacy governs load tolerance and failure character. Limitations include the in-vitro setting, static rather than cyclic or thermomechanical loading, use of a single post-core and crown system and restriction to maxillary central incisors, which may limit generalization to other tooth types and restorative materials. These constraints argue for cautious extrapolation while still supporting the internal validity of the comparative conclusions.

Future work incorporating fatigue and thermal cycling, alternative post-core and crown systems (including fibre posts and all-ceramic reconstructions), and prospective clinical follow-up would strengthen external validity. From an analytic perspective, equivalence or non-inferiority frameworks and Bayesian hierarchical models could help define clinically acceptable margins when only a partial ferrule is achievable, clarifying when a non-uniform collar is functionally adequate versus mechanically compromised. In sum, the present study reinforces a practical hierarchy: a uniform 2-mm ferrule offers performance comparable to conservative crowned approaches, a non-uniform collar confers partial but meaningful protection, and omission of a ferrule carries the greatest risk placing coronal dentin preservation and ferrule creation at the centre of post-endodontic restorative planning.

## Conclusions

Within the limits of this in-vitro study, ferrule configuration was the key determinant of mechanical performance. A uniform 2-mm ferrule preserved fracture resistance comparable to intact crowned and RCT-only crowned teeth and favoured more restorable failure patterns; a non-uniform ferrule offered partial protection but remained inferior to a continuous collar, while no ferrule performed worst. Clinically, prioritize preserving coronal dentin and creating a continuous 2-mm ferrule; when that is not feasible, a partial ferrule is still preferable to none. Confirmation under fatigue/thermomechanical ageing and in prospective clinical studies are warranted to translate these bench findings to long-term outcomes.
